# Analysis of cognitive function trajectories and influencing factors in elderly patients with ischemic stroke: a prospective longitudinal study

**DOI:** 10.3389/fnagi.2026.1733972

**Published:** 2026-01-21

**Authors:** Xinhao Chen, Chengxia Wei, Danli Zhao, Chenmei Zhou, Wenjing Wang, Gendi Lu

**Affiliations:** 1 Department of Neurology, Shanghai University of Traditional Chinese Medicine Affiliated Shanghai Pudong New Area Guangming Hospital of Traditional Chinese Medicine, Shanghai, China; 2 Department of Neurology, Shuguang Hospital Affiliated to Shanghai University of Traditional Chinese Medicine, Shanghai, China; 3 Department of Nursing, Shanghai University of Traditional Chinese Medicine Affiliated Shanghai Pudong New Area Hospital of Traditional Chinese Medicine, Shanghai, China

**Keywords:** cognitive function, influencing factors, ischemic stroke, latentvariables, trajectory

## Abstract

**Background and purpose:**

Accurately defining cognitive function trajectories in elderly ischemic stroke patients is crucial for identifying high-risk groups for cognitive decline, enabling timely personalized interventions to improve long-term outcomes and reduce familial and societal burdens. However, research on the heterogeneity of cognitive trajectories and their underlying causes in elderly Chinese patients with ischemic stroke remains limited. This prospective longitudinal study aimed to detect unique trends in cognitive trajectories among these patients and assess the contributing factors.

**Methods:**

Validated assessment tools were utilized to systematically evaluate cognitive function at various specified intervals. Latent variable growth models were employed to delineate distinct subgroups of cognitive trajectories and to assess the predictive relevance of baseline pathophysiological markers and influencing factors in distinguishing these trajectories.

**Results:**

Cognitive trajectories were categorized into three latent groups: the High Cognitive Function-Slow Decline Group (51%), the Low Cognitive Function-Intermediate Decline Group (34%), and the Low Cognitive Function-Rapid Decline Group (15%). Logistic regression analysis revealed that age, temporal lobe infarction, serum homocysteine levels, National Institutes of Health Stroke Scale score, and Multidimensional Perceived Social Support Scale score, were significant predictors of cognitive function (*p* < 0.05).

**Conclusion:**

Geriatric patients with ischemic stroke have a gradual decline in cognitive function over time. Cognitive change trajectories can be categorized into three latent groups: High Cognitive Function-Slow Decline Group, Low Cognitive Function-Intermediate Decline Group, and Low Cognitive Function-Rapid Decline Group. Healthcare practitioners can develop tailored intervention strategies based on individual patients’ cognitive trajectories and influencing factors to reduce cognitive decline and improve quality of life.

## 1 Introduction

Stroke remains a leading cause of death and disability globally, with its prevalence consistently rising (Liu et al., 2024). Currently, 157 million individuals worldwide have suffered a stroke, with ischemic stroke (IS) accounting for 65.3% of these cases (Collaborators, 2024). In China, IS is a common chronic noncommunicable disease, accounting for about 2 million new cases annually, posing a significant public health threat (Wu et al., 2019). Research indicates that the rise of IS in China predominantly affects the elderly demographic (Chang et al., 2023). The primary causes leading to the incidence of IS among the elderly include China’s swift demographic aging and insufficient health maintenance awareness among elderly patients, culminating in a yearly increase in cerebrovascular disease risk within this demographic (Tu and Wang, 2023). Currently, advancements in technology for the acute phase treatment of IS, such as thrombolysis and thrombectomy, have led to a rise in survival rates and an extension of the survival duration following IS episodes (Huhtakangas et al., 2024). The pathogenesis of IS includes cerebral atherosclerosis and thrombosis, resulting in neuronal damage and disruption of brain connections, which greatly impacts cognitive decline in patients (Candelario-Jalil et al., 2022). Compared to the gradual structural changes in the brain and progressive age-related cognitive decline associated with normal aging, IS induces acute, focal neurological damage characterized by sudden onset and heterogeneity. The pathological responses it triggers include acute excitotoxicity, disruption of the blood–brain barrier, and chronic neuroinflammation with secondary neurodegeneration persisting for months. This is not a simple superposition of age-related decline but rather an interaction that can significantly alter the trajectory and rate of cognitive function in the elderly population (Rost et al., 2022). Post-stroke cognitive impairment (PSCI) is a prevalent consequence subsequent to IS, denoting cognitive deterioration closely linked to or induced by the stroke. It presents as a collection of syndromes that fulfill diagnostic criteria for cognitive impairment within 6 months following the stroke occurrence. 83% of patients exhibit deficits in at least one cognitive domain, whereas 50% manifest impairments across multiple (≥3) domains (Jokinen et al., 2015). A large-scale epidemiological study indicates that experiencing a single stroke event approximately doubles the risk of dementia and accelerates cognitive decline at a faster rate compared to non-stroke peers of the same age, suggesting that stroke significantly hastens the progression of cognitive decline (Pendlebury and Rothwell, 2019). About 30% of IS patients will advance to dementia within 5 years, and approximately 50% of individuals with post-stroke dementia will succumb within 1 year (Zupanic et al., 2021). Cognitive deterioration subsequent to IS presents considerable medical and financial challenges for families and society (Zhang et al., 2025). Studies demonstrate that elderly adults exhibiting significant cerebral vascular fragility are at risk for cognitive loss due to variables such as advanced age and neurodegenerative diseases (Pendlebury and Rothwell, 2019). Further investigation is required to ascertain independent predictors of cognitive impairment following a stroke, including additional predictive blood biomarkers such as apolipoprotein E (ApoE) (El et al., 2023). Therefore, cognitive changes in elderly IS patients do not follow a single, gradual trajectory but instead exhibit diverse patterns driven by distinct factors. Identifying these trajectories and their underlying drivers enables the recognition of high-risk patients, facilitating early intervention to halt accelerated cognitive decline.

Nonetheless, contemporary studies on alterations in cognitive function in elderly Chinese IS patients exhibit numerous drawbacks. Although studies reveal a significant prevalence of cognitive impairment in IS patients, the progression of cognitive function and the factors influencing it in the senior IS demographic remain ambiguous (El et al., 2023). Cognitive function experiences a dynamic evolution following the start of IS, demonstrating complexity and variability in its patterns of change. Secondly, previous research suggests that the majority of studies on the cognitive function of IS patients depend on cross-sectional surveys. Although these offer average alterations at designated time intervals or among groups, they are deficient in temporal dimensions necessary for examining cognitive change patterns at both individual and group levels (Chen and Lu, 2022). Furthermore, current longitudinal studies on cognitive decline primarily utilize linear mixed-effects models. Although these models integrate random factors to concurrently assess individual variations in baseline cognitive capabilities and subsequent decline rates, they neglect to consider potentially varied developmental trajectories within populations (Li et al., 2020). Research utilizing Latent Growth Curve Models (LGCM) helps elucidate the developmental patterns of cognitive function trajectories. LGCM enhances the analysis by explicitly modeling individuals’ beginning levels and developmental rates as latent variables, thereby incorporating causal linkages within trajectories. Moreover, chronic illness trajectory theory posits that disease progression does not adhere to a singular, uniform pathway but rather displays nonlinear, variable trajectories. This variation exists both across and within groups, requiring individualized prognosis evaluation and informed decision-making (Xu et al., 2025).

This prospective longitudinal study utilizes LGCM to examine cognitive alterations and their determinants in elderly Chinese patients with IS, addressing the constraints of current research on cognitive function in this demographic and the ambiguity regarding chronic illness trajectories. This method has considerable consequences for precisely identifying high-risk groups and formulating tailored intervention measures. Investigating cognitive performance in elderly IS patients is of particular importance and urgency given China’s distinct social, cultural, and healthcare contexts.

## 2 Materials and methods

### 2.1 Study sample

This study utilized convenience sampling to recruit elderly IS patients admitted to neurology wards at three hospitals in Shanghai, China, from January 2023 to December 2024. The inclusion criteria were: fulfillment of the diagnostic criteria (Martha et al., 2023) for IS (age ≥ 60 years; signed informed permission; onset ≤ 14 days). Exclusion criteria encompassed: a history of traumatic brain damage or intracerebral hemorrhage; concurrent central nervous system illnesses (e.g., intracranial space-occupying lesions, Parkinson’s disease, epilepsy); assessment by a neurology team confirming a life expectancy of less than 6 months (e.g., patients with severe consciousness impairment, advanced malignant tumors, end-stage cardiac/pulmonary/renal failure, or undergoing tracheal intubation or tracheostomy);coexisting psychiatric conditions, including schizophrenia, moderate to severe mental problems, or dementia; Administration of drugs influencing mental status within 3 months preceding stroke onset or during follow-up; death, rejection of follow-up, or voluntary departure from the study. The Ethics Committee of Shanghai University of Traditional Chinese Medicine accepted this study (Approval No.: 2023–1,317-84-01).

### 2.2 Cognitive function assessment

This study used the Montreal Cognitive Assessment Scale (MoCA) as the cognitive function assessment tool, which appraises seven cognitive domains: visuospatial and executive function, name, attention, language, abstraction, delayed recollection, and orientation, totaling 30 items. Correct responses are awarded 1 point, whereas erroneous ones receive 0 points. The cumulative score varies from 0 to 30 points. Participants possessing fewer than 12 years of schooling are awarded an additional point on their exam scores. Elevated scores signify superior cognitive function, and scores below 26 denote cognitive disability. This scale is a globally acknowledged complete neurological cognitive assessment instrument (Jia et al., 2021). The assessment timepoints for cognitive function were determined based on the four stages of the stroke illness trajectory (Kirkevold, 2002): onset stage, initial recovery stage, sustained recovery stage, and semi-stable stage. These correspond to ≤48 h following the IS event (T1), 1 month (T2), 3 months (T3), and 6 months (T4). All subsequent evaluations were performed at the hospital’s stroke follow-up clinic.

### 2.3 General information collection

This study’s survey instruments, informed by prior literature (Chen and Lu, 2022), encompassed a general information questionnaire that addressed demographic variables (age, gender, years of education), vascular factors [history of hypertension, diabetes, hyperlipidemia, atrial fibrillation, coronary heart disease, body mass index (BMI), alcohol consumption history, smoking history, statin usage, carotid plaque history], stroke-related factors (history of stroke, location of temporal lobe infarction, timely thrombolytic therapy), and serum biomarker levels [serum homocysteine (Hcy), ApoE, C-reactive protein (CRP)]. Blood samples were collected via regular medical protocols, preserved in tissue banks, and subsequently analyzed. Smoking history is defined as cumulative or continuous smoking over a duration of at least 6 months during one’s lifetime, involving a minimum of four cigarettes per week. Drinking history is defined as current or past ethanol consumption of at least 140 grams per week (or 70 grams per week for females). Neurological disability and stroke severity were evaluated utilizing the National Institute of Health Stroke Scale (NIHSS) (Martha et al., 2023). The Multidimensional Scale of Perceived Social Support (MSPSS) was employed to evaluate social support levels across three dimensions: familial support, friendship support, and additional support. The 12-item scale produces a cumulative score between 12 and 84 points. Elevated scores signify enhanced felt social support. Scores are classified into three tiers: 12–36 points signify low levels, 37–60 points denote moderate levels, and 61–84 points represent high levels (Lee et al., 2023). The Barthel Index was employed to evaluate patients’ activities of daily living and functional impairment (Achilike et al., 2020).

### 2.4 Sample size

The sample size for this study was estimated using the Kendall method (Dufey, 2020). The sample size should be 10–20 times the number of study variables. With 22 variables in this study, an additional 10% sample size was added to account for potential data invalidation due to follow-up loss. The final calculated required sample size ranged from 242 to 484 cases. This study enrolled 345 patients, with 330 (95.7%) ultimately completing follow-up. Among these, 8 patients were lost to follow-up for various reasons, 3 died due to other unforeseen events, and 4 refused further follow-up.

### 2.5 Quality control

Patients with IS are managed by doctors according to defined guidelines, utilizing uniform therapeutic protocols that encompass antihypertensive therapy, cerebral edema reduction, anticoagulation, thromboprophylaxis, and additional therapies and drugs. Discharge medications comprise standard antihypertensive and lipid-lowering agents. Patients receive monthly outpatient follow-ups for six months following discharge, and drugs that impact cognitive function are excluded. All study methods were executed by research team members who received standardized training.

MoCA assessments were conducted in full accordance with the scale’s usage rules, based on a rigorous study design and early exploratory analysis. To guarantee assessment quality, all evaluators underwent comprehensive training encompassing the scale’s theoretical framework, established protocols, scoring standards, and prevalent scenario management strategies. Subsequent to training, simulated evaluations and inter-rater reliability assessments validated each member’s proficiency in test administration, thereby guaranteeing standardized data gathering and comparable outcomes. When patients could not attend follow-up appointments or cognitive evaluations within the designated timeframe for any reason, remote video follow-ups were performed. Instances of lost contact or unavailability of cooperation for follow-up were classified as loss to follow-up to mitigate bias from sample attrition.

### 2.6 Statistical analysis

Continuous variables are presented as mean (standard deviation) or median (interquartile range). Categorical variables were presented as numbers (percentages). The LGCM was constructed using Mplus software version 8.3(Muthén & Muthén Inc., Los Angeles, US), and its goodness-of-fit was assessed to determine whether heterogeneity existed in the cognitive development trajectories of elderly IS patients. LGCM model evaluation metrics included: (1) Information indices: Akaike Information Criterion (AIC), Bayesian Information Criterion (BIC), adjusted BIC (αBIC), and Entropy. Among these, entropy is used to evaluate the precision of model classification, ranging from 0 to 1. A higher value indicates better separation between the latent categories identified by the model and more certain classification; (2) Test statistics: Likelihood Ratio Test (LRT) and Bootstrapped Likelihood Ratio Test (BLRT) to determine the optimal number of groups. Statistically significant differences (*p* < 0.05) in LRT and BLRT values indicate that the “n-class” model outperforms the “n-1-class” model. AIC, BIC, and αBIC measure relative model fit, where lower values indicate better fit. Entropy values maximize the number of model categories, with values closer to 1 representing more precise classification (Xu et al., 2025). In LGCM, the intercept (i) and slope (s) each have two parameters: mean and variance. The intercept mean describes the average initial state of individuals, while the slope mean reflects the average growth rate between time points. The significance of the intercept and slope means respectively, represents differences in initial levels and growth rates. For univariate analysis, normally distributed and homoscedastic quantitative data were compared using analysis of variance (ANOVA). For skewed quantitative data, comparisons were performed using nonparametric rank-sum tests. Categorical data comparisons utilized chi-square tests. Logistic regression analysis was conducted with latent categories as dependent variables, incorporating statistically significant variables from univariate analysis to identify independent factors. *p* < 0.05 was considered statistically significant.

The statistical analysis in this study was conducted in two phases. First, univariate analysis was performed to preliminarily screen all potential predictors, aiming to identify candidate variables potentially associated with cognitive trajectory categories. The screening threshold was set at *p* < 0.05, and this phase constituted exploratory analysis. Subsequently, the variables identified through univariate analysis were incorporated into a multinomial logistic regression model to clarify the independent associations between each factor and different cognitive trajectory categories. To control for multiple comparisons, statistical significance in the logistic regression model was strictly interpreted using Bonferroni correction during this validation phase, with the correction threshold determined based on the number of comparisons among independent variables in the final model.

## 3 Results

### 3.1 Basic characteristics of the study sample

**Figure 1:**
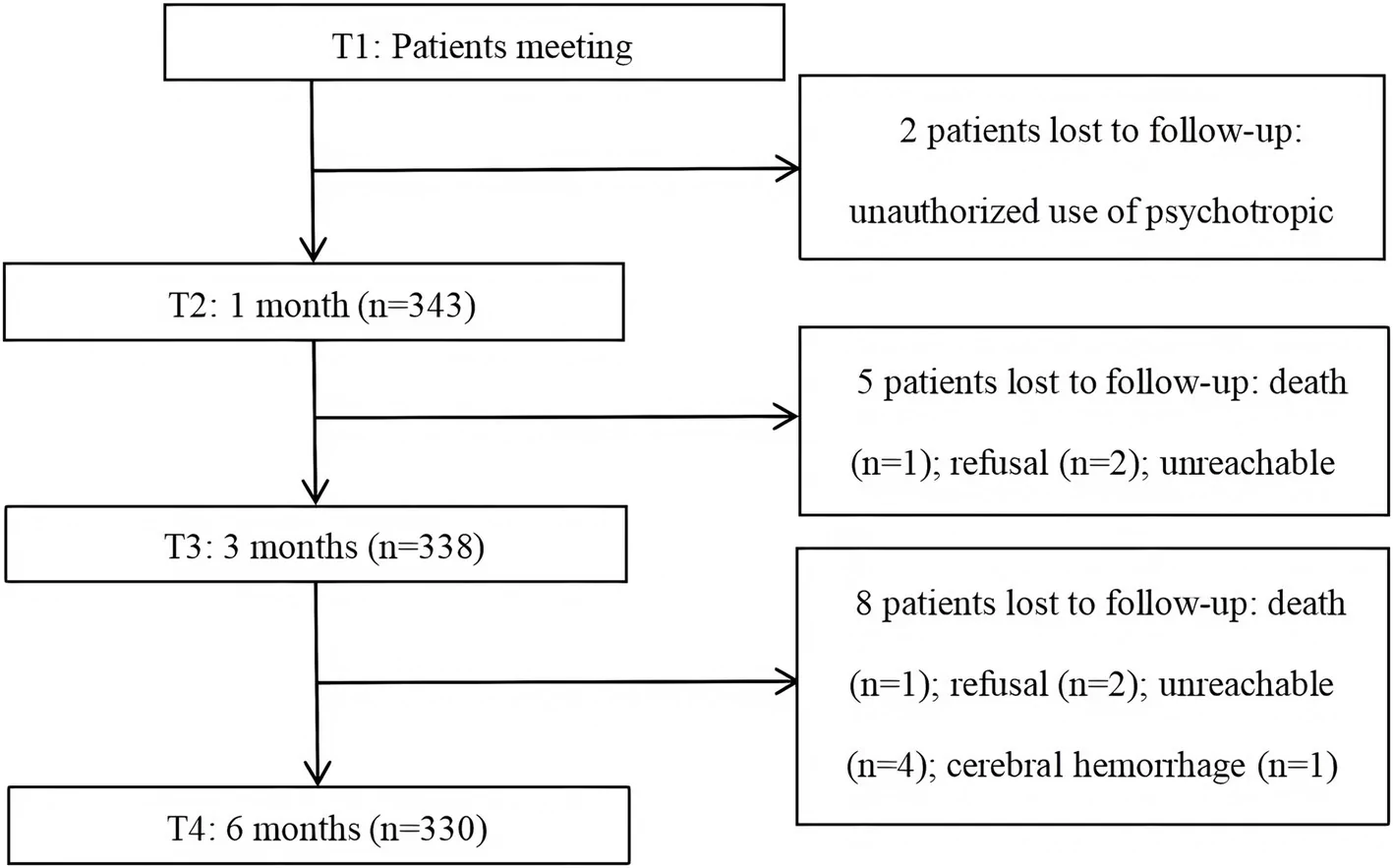
General characteristics of the 330 enrolled patients.

A total of 345 elderly patients with IS were enrolled within 48 h of the IS event (T1). During the 6-month follow-up period, 2 patients were lost to follow-up at 1 month (T2), 5 at 3 months (T3), and 8 at 6 months (T4). Ultimately, 330 patients completed the entire study, resulting in a loss to follow-up rate of 4.3%. Analysis of non-response bias revealed no statistically significant differences between non-responders (*n* = 15) and follow-up completers (*n* = 330) in baseline cognitive function assessments and other characteristics. Detailed data are presented in the enrollment flowchart (Figure 1). General characteristics of the 330 enrolled patients are detailed in Table 1.

**Table 1:** General characteristics of study subjects (*n* = 330).

Variable	Group	n(%)
Gender	Male	236 (71.5)
	Female	94 (28.5)
Age	≤75 years	238 (72.1)
	>75 years	92 (27.9)
MoCA score (T1)	<26 points	139 (42.1)
	≥26 points	191 (57.9)
Smoking history	Yes	83 (25.2)
Alcohol consumption history	Yes	28 (8.5)
History of diabetes	Yes	131 (39.7)
History of hypertension	Yes	269 (81.5)
History of atrial fibrillation	Yes	14 (4.2)
Years of education	≤12 years	169 (51.2)
	>12 years	161 (48.8)

### 3.2 Identifying latent categories of cognitive function trajectories in elderly IS patients

A heterogeneity analysis was performed on the cognitive function scores of 330 elderly IS patients who completed all four time-point assessments. LGCM was employed for model fitting, with particular results delineated in Table 2. This study derived between one to four models, commencing with a singular model type and progressively augmenting the number of kinds in the LCGM to ascertain the model that most accurately represents the data. As the variety of types expanded, the values of AIC, BIC, and **α**BIC consistently diminished. Upon keeping four kinds, the LRT and BLRT values had little statistical significance, however Entropy demonstrated a considerable drop. Retaining three kinds produced the lowest BIC value, with the AIC being less than that of the two-type model. The Entropy value above 0.9, and both the LRT and BLRT were statistically significant (*p* < 0.05). The study picked the model that retains three trajectory types. The analysis of the average probability for each latent category revealed values beyond 90%, demonstrating the model’s stability and reliability.

**Table 2:** Model fitting results for cognitive trajectory latent categories in elderly IS patients.

Model	AIC	BIC	αBIC	Entropy	LRT (P)	BLRT (P)	Category probability
1	4813.076	4828.272	4815.584				
2	4100.830	4127.423	4105.219	0.994	<0.001	<0.001	0.85/0.15
3	**3779.243**	**3817.234**	**3785.514**	**0.979**	**<0.001**	**0.002**	**0.15/0.51/0.34**
4	3777.270	3826.658	3785.422	0.865	0.078	0.429	0.15/0.37/0.14/0.34

AIC, Akaike Information Criterion; BIC, Bayesian Information Criterion; αBIC, Adjusted Bayesian Information Criterion; LRT, Likelihood Ratio Test; BLRT, Bootstrap-based Likelihood Ratio Test; P, *p*-value. Bolded values represent the latent categories selected in this study.

### 3.3 Naming and identifying subgroups of cognitive function trajectories in elderly IS patients

**Figure 2:**
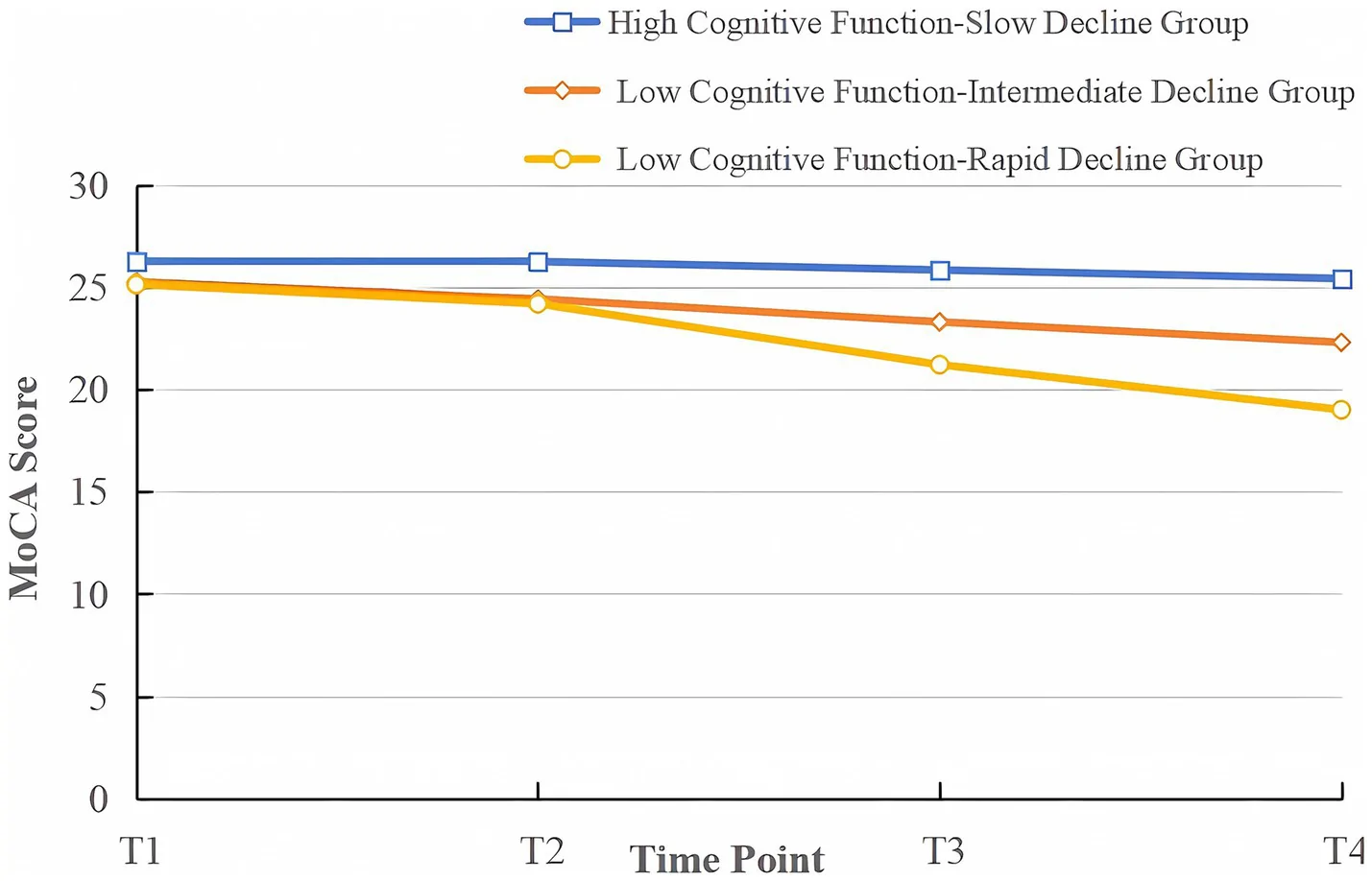
Patients in category 1 exhibited a mean cognitive function score of 26.324, indicating a progressive reduction over time (*s* = −0.152, *p* < 0.001). Patients in ategory 2 exhibited a mean cognitive function score of 25.005, indicating a moderate deterioration with time (s = −0.482, *p* < 0.001). Patients in category 3 exhibited a mean cognitive function s core of 24.988, demonstrating a significant deterioration over time (s = −1.046, *p* < 0.001).

According to the results of the latent class analysis, cognitive function trajectory maps were constructed, with the vertical axis denoting cognitive function scores in elderly IS patients and the horizontal axis representing time points from T1 to T4, as illustrated in Figure 2. The three cognitive function trajectory groups identified by this study were automatically recognized and assigned by LGCM based on the patterns of MoCA score changes across four time points from T1 to T4. The model first identifies the three most representative cognitive change trajectories within the data. It then calculates the posterior probability of each patient’s assignment to these categories based on the match between their four individual scores and these typical trajectories. Patients are ultimately assigned to the category with the highest probability. This grouping is based on the individual’s complete longitudinal change pattern, not a single score at any one time point.

According to the beginning values, slopes, and trends in MoCA score variations at various time intervals along the three developmental trajectories, they are designated as follows: Category 1: Patients in this category demonstrated a high baseline cognitive function score (i = 26.324), with a mean considerably exceeding that of Categories 2 and 3. Their scores indicated normal cognitive function, although exhibited a gradual deterioration over time (s = −0.152, *p* < 0.001). The group is designated as the High Cognitive Function-Slow Decline Group (C1). Category 2: Patients in this cohort demonstrated a diminished baseline cognitive function score (i = 25.005) and displayed a moderate reduction over time (s = −0.482, *p* < 0.001). This cohort is designated as the Low Cognitive Function-Intermediate Decline Group (C2). Category 3: Patients in this cohort demonstrated a low baseline cognitive function score (i = 24.988) and a swift deterioration over time (s = −1.046, *p* < 0.001), designated as the Low Cognitive Function-Rapid Decline Group (C3). Detailed comparisons of cognitive function scores across the three groups at different time points are presented in Table 3. The MoCA score represents the average characteristic of each group. The actual values of each patient within a group fluctuate around this average, meaning that all patients within a group have values distributed near the mean.

**Table 3:** Cognitive function scores comparison across time points for three groups.

Group	Number of cases (*n* = 330)	T1	T2	T3	T4
C1	169	26.26 ± 2.86	26.24 ± 3.02	25.83 ± 3.41	25.41 ± 3.72
C2	111	25.26 ± 2.89	24.41 ± 3.02	23.29 ± 3.39	22.29 ± 3.70
C3	50	25.12 ± 2.85	24.18 ± 3.01	21.20 ± 3.39	19.00 ± 3.71
F	-	5.470	17.606	55.374	103.542
P	**-**	**0.005**	**<0.001**	**<0.001**	**<0.001**

C1, High Cognitive Function-Slow Decline Group; C2, Low Cognitive Function-Intermediate Decline Group; C3, Low Cognitive Function-Rapid Decline Group; F: Analysis of variance; P, *P*-value. Bolded values indicate statistically significant differences.

### 3.4 Univariate analysis of cognitive trajectory categories in elderly IS patients

This study analyzed features of elderly IS patients across three different cognitive trajectory types. The results indicated statistically significant differences in age, history of coronary heart disease, temporal lobe infarction, years of education, Hcy levels, MSPSS score, and NIHSS score (*p* < 0.05). The findings of the detailed analysis are reported in Table 4.

**Table 4:** Univariate analysis of cognitive trajectory subgroups in elderly IS patients.

Item	Category	Group			Z/χ^2^/*F* value	*p* value
		C1 (*n* = 169)	C2 (*n* = 111)	C3 (*n* = 50)		
Gender, *n* (%)					2.470^a^	0.291
	Male	116 (68.6)	80 (72.1)	40 (80.0)		
	Female	53 (31.4)	31 (27.9)	10 (20.0)		
Smoking history, *n* (%)					1.470^a^	0.480
	Yes	38 (22.5)	30 (27.0)	15(30.0)		
	No	131 (77.5)	81 (73.0)	35 (70.0)		
Past ethanol intake, *n* (%)					2.353^a^	0.308
	Yes	11 (6.5)	13 (11.7)	4(8.0)		
	No	158(93.5)	98 (88.3)	46 (92.0)		
Diabetes, *n* (%)					0.792^a^	0.673
	Yes	71 (42.0)	41 (36.9)	19 (38.0)		
	No	98 (58.0)	70 (63.1)	31 (62.0)		
Hypertension, *n* (%)					3.132^a^	0.209
	Yes	132 (78.1)	96 (86.5)	41(82.0)		
	No	37 (21.9)	15 (13.5)	9 (18.0)		
Hyperlipidemia, *n* (%)					0.658^a^	0.719
	Yes	72 (42.6)	42 (37.8)	21(42.0)		
	No	97 (57.4)	69 (62.2)	29 (58.0)		
Coronary heart disease, *n* (%)					6.907^a^	**0.032**
	Yes	21 (12.4)	15 (13.5)	14(28.0)		
	No	148 (87.6)	96 (86.5)	36 (72.0)		
Atrial fibrillation, *n* (%)					4.927^a^	0.085
	Yes	6(3.6)	3 (2.7)	5(10.0)		
	No	163 (96.4)	108 (97.3)	45(90.0)		
Previous stroke, *n* (%)					4.360^a^	0.113
	Yes	80 (47.3)	55 (49.5)	32(64.0)		
	No	89 (52.7)	56 (50.5)	18 (36.0)		
Temporal lobe infarction, *n* (%)					36.973^a^	**<0.001**
	Yes	11 (6.5)	23 (20.7)	21 (42.0)		
	No	18(93.5)	88 (79.3)	29(58.0)		
Thrombolysis performed this time, *n* (%)					2.992^a^	0.224
	Yes	21 (12.4)	11(9.9)	2(4.0)		
	No	148(87.6)	100 (90.1)	48 (96.0)		
History of statin use, *n* (%)					1.073^a^	0.585
	Yes	159 (94.1)	101 (91.0)	47 (94.0)		
	No	10 (5.9)	10 (9.0)	3(6.0)		
Education, *n* (%)					13.034^a^	**0.001**
	≥12 years	97 (57.4)	49 (44.1)	15 (30.0)		
	<12 years	72 (42.6)	62 (55.9)	35 (70.0)		
Presence of carotid plaque, *n* (%)					3.642^a^	0.162
	Yes	132 (78.1)	91 (82.0)	45(90.0)		
	No	37 (21.9)	20 (18.0)	5 (10.0)		
Age, years	-	69 (64, 73)	73 (67, 78)	78 (70, 81)	45.562^b^	**<0.001**
CRP, mg/L	-	0.16 (0.08, 0.39)	0.20 (0.08, 0.67)	0.16 (0.08, 0.59)	1.638^b^	0.441
Hcy, μmol/L	-	12.7 (9.9, 14.8)	14.6 (12.0, 19.2)	17.5 (11.9, 25.2)	30.644^b^	**<0.001**
ApoE, mg/dL	-	4.22 ± 1.36	4.22 ± 1.32	4.51 ± 1.28	1.008^c^	0.366
NIHSS, score	-	3(2, 4)	4(3, 6)	5(4, 8)	47.033^b^	**<0.001**
BMI, kg/m^2^	-	24.4 ± 3.2	24.2 ± 3.3	23.4 ± 3.0	1.885^c^	0.153
Barthel Index, score	-	74.6 ± 14.6	75.4 ± 13.3	71.1 ± 17.8	1.510^c^	0.223
MSPSS, score	-	60.6 ± 17.5	55.2 ± 18.5	51.5 ± 19.0	6.115^c^	**0.002**

P, *P*-value; CRP, C-reactive protein; Hcy, homocysteine; ApoE, Apolipoprotein E; NIHSS, National Institutes of Health Stroke Scale; BMI, Body Mass Index; MPSSS, Multidimensional Perceived Social Support Scale. C1, High Cognitive Function-Slow Decline Group; C2, Low Cognitive Function-Intermediate Decline Group; C3, Low Cognitive Function-Rapid Decline Group. This table presents univariate analysis. *p*-values are used for preliminary exploratory variable analysis and screening. Final conclusions are based on results from the multivariate regression model after Bonferroni correction. Bolded values indicate statistically significant differences.

a Chi-square test.

b Nonparametric rank-sum test.

c Analysis of variance.

### 3.5 Multivariate analysis of cognitive trajectory latent categories in elderly IS patients

This study utilized multivariate logistic regression analysis, with latent categories of cognitive function trajectories in elderly IS patients as the dependent variable and statistically significant covariates from univariate analysis as independent variables. The “Low Cognitive Function-Intermediate Decline Group” was designated as the reference group. The coding technique for independent variables is presented in Table 5.

**Table 5:** Variable coding for logistic regression analysis.

Variable	Coding method
Education	Years of education < 12 = 0, Years of education ≥12 = 1
Coronary heart disease	No = 0, Yes = 1
Temporal lobe infarction	No = 0, Yes = 1
Age	Raw value
Hcy	Raw value
NIHSS	Raw value
MSPSS	Raw value

Hcy, homocysteine; NIHSS, National Institutes of Health Stroke Scale; MPSSS, Multidimensional Perceived Social Support Scale.

Multivariate logistic regression findings indicated that, in comparison to the Low Cognitive Function-Intermediate Decline Group (C2), the High Cognitive Function-Slow Decline Group (C1) exhibited: advancing age (OR = 1.116, 95% CI: 1.062–1.172, *p* < 0.001); increased serum Hcy concentrations (OR = 1.126, 95% CI: 1.054–1.203, *p* < 0.001); and elevated NIHSS score (OR = 1.347, 95% CI: 1.171–1.549, *p* < 0.001) as independent risk factors for cognitive decline. Elevated MSPSS scores (OR = 0.971, 95% CI: 0.954–0.987, *p* = 0.001) served as protective factors. In comparison to the Low Cognitive Function-Intermediate Decline Group (C2), the Low Cognitive Function-Rapid Decline Group (C3) exhibited that temporal lobe infarction (OR = 5.125, 95% CI: 2.031–12.933, *p* = 0.001) was independent risk factors for accelerated cognitive decline, as outlined in Table 6.

**Table 6:** Univariate analysis of cognitive trajectory subgroups in elderly IS patients.

Dependent variable	Independent variable	β	SE	Wald χ^2^	*P* value	OR(95%CI)
C1 vs. C2	Constant	−9.317	1.867	24.907	0.001	–
	Age	0.109	0.025	19.149	**<0.001**	1.116(1.062–1.172)
	Education	−0.531	0.303	3.059	0.080	0.588(0.324–1.066)
	Coronary heart disease	0.536	0.415	1.671	0.196	1.709(0.758–3.854)
	Hcy	0.119	0.034	12.544	**<0.001**	1.126(1.054–1.203)
	Temporal lobe infarction	1.437	0.492	8.511	0.004	4.207(1.602–11.043)
	NIHSS	0.298	0.071	17.485	**<0.001**	1.347(1.171–1.549)
	MSPSS	−0.030	0.009	11.755	**0.001**	0.971(0.954–0.987)
C3 vs. C2	Constant	−5.255	2.214	5.634	0.018	-
	Age	0.052	0.029	3.291	0.070	1.054(0.996–1.115)
	Education	−0.925	0.432	4.584	0.032	0.397(0.170–0.925)
	Coronary heart disease	0.576	0.462	1.553	0.213	1.778(0.719–4.395)
	Homocysteine	0.031	0.017	3.295	0.070	1.032(0.998–1.067)
	Temporal lobe infarction	1.634	0.472	11.970	**0.001**	5.125(2.031–12.933)
	NIHSS	0.166	0.073	5.085	0.024	1.180(1.022–1.363)
	MSPSS	−0.022	0.011	4.005	0.045	0.978(0.957–0.999)

C1, High Cognitive Function-Slow Decline Group; C2, Low Cognitive Function-Intermediate Decline Group; C3, Low Cognitive Function-Rapid Decline Group; β, Regression coefficient; SE, Standard error; Wald χ^2^, Wald chi-square value; OR, Odds ratio; 95% CI, 95% confidence interval; P, *P*-value; Hcy, homocysteine; NIHSS, National Institutes of Health Stroke Scale; MSPSS, Multidimensional Perceived Social Support Scale. Bolded values indicate statistically significant differences. To control for multiple comparisons, the Bonferroni correction was applied to *p*-values, with the corrected significance threshold set at 0.00357. Bolded values indicate statistically significant differences.

## 4 Discussion

This study illustrates that all cognitive function trajectories in elderly IS patients display a downward tendency. The initial MoCA score within 48 h following the IS event was 25.75 ± 2.93 points, signifying extensive cognitive impairment in this population that deteriorates with time. The clinical foundation for cognitive deterioration in elderly IS patients entails chronic, progressive network dysfunction that disseminates from acute, focal cerebral vascular occlusion to the entire brain. The process initiates with direct neuronal death and disconnection, succeeded by prolonged neuroinflammation and oxidative stress-related damage, simultaneously exacerbating neurodegenerative consequences (Rost et al., 2022). Consequently, healthcare professionals must emphasize the evaluation of cognitive function in elderly IS patients, apply classified treatment according to assessment outcomes, and deliver focused therapies to mitigate cognitive decline in this demographic.

This study utilized Latent Growth Curve Modeling (LGCM) to delineate three latent categories of cognitive decline trajectories among older individuals with IS: “High Cognitive Function-Slow Decline Group (C1),” “Low Cognitive Function-Intermediate Decline Group (C2),” and “Low Cognitive Function-Rapid Decline Group (C3).” This suggests that cognitive decline trajectories in elderly IS patients demonstrate a general decreasing tendency with notable group-specific variances. Studies demonstrate that IS occurrences in elderly people exacerbate brain injury, inflammation, and neurological deficits, hastening the advancement of neurodegenerative processes and resulting in persistent cognitive deterioration (Lo et al., 2024). The variability in cognitive trajectories among elderly IS patients results from a combination of factors, including the severity and location of the initial brain injury, individual cognitive reserve, management of systemic vascular risk factors, and the robustness of the psychosocial support environment (El et al., 2023). This study suggests that the key factors contributing to the gradual cognitive decline in the High Cognitive Function-Slow Decline Group (C1) are likely lower neurological impairment and elevated baseline cognitive performance. The fast cognitive deterioration in the Low Cognitive Function-Intermediate Decline Group (C3) may stem from a restricted cognitive reserve coupled with impairment to essential brain areas. The low-cognitive-level-moderate-decline group (C2) encompasses a diverse population in a transitional state. The findings of this study have substantial clinical consequences, highlighting the need for early comprehensive assessment of elderly IS patients. This facilitates accurate identification of people in the Low Cognitive Function-Rapid Decline Group (C3) undergoing accelerated cognitive decline, permitting targeted, multi-faceted, tailored therapies to disrupt the detrimental cycle and enhance prognosis.

Assessments of cognitive function in elderly IS patients reveal substantial variations at distinct time intervals. The disease progression of IS comprises four stages: the onset stage, the initial recovery stage, the sustained recovery stage, and the semi-stable stage (Kirkevold, 2002). The initial measurement takes place within 48 h following the IS event (T1), signifying the onset stage. This stage involves direct damage from the infarct, leading to decreased brain function and a significant loss in cognitive ability relative to baseline (El et al., 2023). In this study, both the Low Cognitive Function-Intermediate Decline Group (C2) and the Low Cognitive Function-Rapid Decline Group (C3) demonstrated cognitive function scores beneath the normative range of the MoCA cognitive function standard at the T1 time point. The cognitive function levels in these two groups may correlate with the brain’s inflammatory edema response. The recovery stage consists of the initial recovery stage at 1 month and the sustained recovery stage at 3 months following the commencement of the IS. Patients in the initial recovery stage demonstrate progressively stable cognitive function as acute pathological responses subside, although complete cognitive return to pre-illness levels may not be achievable.

This study revealed that the cognitive function levels of the High Cognitive Function-Slow Decline Group (C1) and the Low Cognitive Function-Intermediate Decline Group (C2) aligned with trends indicative of the initial recovery stage, whereas the Low Cognitive Function-Rapid Decline Group (C3) demonstrated a significant decline. At the sustained recovery stage (T3), the cognitive function level of the Low Cognitive Function-Rapid Decline Group (C3) exhibited a substantial decrease compared to both the onset stage (T1) and the initial recovery stage (T2). This may be associated with diminished cognitive reserve and pronounced IS disease severity during this timeframe, hindering neuronal compensation for injured brain regions and impeding neuroplasticity-related physiological responses, resulting in ongoing cognitive decline (Yan et al., 2025). The measuring time point T4 during the semi-stable stage occurred six months post-IS beginning. At this stage, elderly IS patients exhibited a gradual deterioration in cognitive function resulting from the ongoing advancement of cerebrovascular lesions and subsequent neurodegeneration. Severe instances advanced to post-stroke cognitive impairment (Lo et al., 2022; Yan et al., 2025). The persistent deterioration in cognitive performance across all groups in this study corresponds with the trend noted during the semi-stable stage.

Initial study found age, serum Hcy levels, temporal lobe infarction, NIHSS score, and MSPSS score as factors affecting cognitive change. Age constitutes an immutable factor influencing cognitive impairment in elderly IS patients, corroborating prior research (Rost et al., 2022). Significantly diminished cerebral vascular elasticity and contractility exacerbate cerebral ischemia and hypoxia during IS episodes, resulting in amyloid deposition, disruption of hippocampal connections, and accelerated cognitive decline (Shen et al., 2020). Furthermore, aging is positively associated with the prevalence of chronic diseases. Chronic disease-related tiredness increases the likelihood of cognitive deterioration (Weaver et al., 2021). Consequently, prompt cognitive training should be administered to elderly IS patients to postpone cognitive deterioration.

In this study, among the Low Cognitive Function-Intermediate Decline Group (C2) compared to the Low Cognitive Function-Rapid Decline Group (C3), longer years of education were associated with a reduced risk of rapid cognitive decline (OR = 0.397). Although this association did not reach statistical significance after multiple comparison correction, its effect size remains noteworthy. Although educational attainment does not guarantee protection against neurological disorders, it offers a mitigating influence on associated functions. Individuals with equivalent levels of brain injury demonstrate enhanced cognitive resilience (Li et al., 2020). The learning process consistently engages the brain, strengthening neuronal signaling pathways and supporting brain plasticity, which encompasses the ability to adapt and change (Barth et al., 2025). Research demonstrates that the trajectory of cognitive decline in elderly IS patients is linked to cognitive reserve, with a negative correlation between the rate of decline and cognitive reserve (Delgado et al., 2022). Consequently, education and continuous cognitive engagement are essential for maintaining cognitive reserve. Ongoing information acquisition and improved learning strategies can preserve cognitive function in elderly individuals and postpone cognitive deterioration. This finding suggests that future large-scale studies should further validate this association and explore the potential of educational strategies to increase cognitive reserve as a means of counteracting post-stroke cognitive decline.

The investigation revealed that serum Hcy levels were diminished in the High Cognitive Function-Slow Decline Group (C1) relative to the Low Cognitive Function-Intermediate Decline Group (C2) (OR = 1.126, 95% CI: 1.054–1.203, *p* < 0.001). Elevated serum homocysteine levels are associated with an increased risk of IS. Elevated levels may induce vascular endothelial damage and facilitate atherosclerotic plaque development (Pinzon et al., 2023). Moreover, research suggests that blood Hcy functions as an agonist for NMDA-type glutamate receptors. Overactivation of NMDA receptors leads to excitotoxicity, resulting in excessive calcium influx and subsequent cognitive impairment (Deep et al., 2024; Filipova et al., 2025). Beydoun et al. discovered that cognitive performance markedly deteriorated with elevated blood Hcy levels (Beydoun et al., 2024). B vitamins, as essential cofactors in the breakdown of Hcy in human serum, demonstrate an inverse relationship with serum Hcy levels. Studies suggest that patients with elevated baseline Hcy levels (≥12.1 μmol/L) may experience a trend toward reduced cognitive decline with B vitamin supplementation (folate, B6, B12) (Wang et al., 2022). Consequently, healthcare practitioners must monitor elderly IS patients with serum Hcy levels of 10 μmol/L or higher. Considering individual cognitive performance, it is advisable to supplement with B-vitamin-rich foods, such as green vegetables, while restricting the consumption of red meat and processed foods.

The temporal lobe serves as the speech center within the brain’s cognitive domain. The anatomical integrity of the temporal lobe is essential for cognitive function. Vascular infarction in this area can immediately result in executive dysfunction, poor speech fluency, and memory problems in patients. Severe instances may demonstrate spatial disorientation and unregulated excessive actions (Schmahmann and Sherman, 1998; Wang et al., 2021). This study demonstrates that temporal lobe infarction is the predominant risk factor for cognitive loss among all cohorts of elderly IS patients. Consequently, for elderly IS patients exhibiting temporal lobe infarction on admission imaging, prompt treatment of temporal lobe ischemia should be prioritized. Therapeutic interventions, including thrombolysis and vascular stenting, are advised to reduce irreversible cognitive deficits resulting from temporal lobe ischemia.

This study employed the NIHSS score to assess neurological impairment and stroke severity in elderly IS patients. Results demonstrated that the NIHSS score was lower inhe High Cognitive Function-Slow Decline Group (C1) compared to the Low Cognitive Function-Intermediate Decline Group (C2) (OR = 1.347, 95% CI: 1.171–1.549, *p* < 0.001). This suggests that greater neurological damage is associated with more significant cognitive loss, in agreement with the findings of Esmael et al. (2021). This scale evaluates levels of consciousness, motor function, sensory perception, verbal abilities, visual capacity, and neglect. Elevated scores signify larger infarct regions or infarcts situated in more vital functional areas, leading to greater cerebral tissue damage (Tuxunjiang et al., 2025). This measure disproportionately emphasizes language function and is largely unresponsive to significant spatial neglect and attention deficits often resulting from strokes in the non-dominant hemisphere (Lyden et al., 1994). Consequently, risk classification utilizing acute-phase NIHSS scores should be augmented by standardized neuropsychological evaluations for all stroke survivors to facilitate early detection and treatment for cognitive deficits.

This study utilized the MSPSS score to assess psychosocial contextual factors in elderly IS patients, demonstrating that social support serves as a protective factor against cognitive deterioration. Studies demonstrate that insufficient social support serves as a chronic psychological stressor, specifically exacerbating loneliness in older persons, which, when sustained, may result in increased cortisol levels (Adam et al., 2006). Increased cortisol levels have neurotoxic effects on the hippocampus, including neuronal shrinkage, apoptosis, and reduced neurogenesis, which directly hinders memory and learning capabilities (Ouanes and Popp, 2019). Research indicates that elevated social support is associated with enhanced cognitive function and reduced rates of cognitive deterioration (Mogic et al., 2023). This protective effect may be facilitated by various mechanisms: robust social support can mitigate psychological stress, thereby alleviating negative emotions such as depression and anxiety (Chu et al., 2025); concurrently, it can foster healthy behaviors by promoting healthy lifestyles and adherence to treatment (Dogan et al., 2025). For elderly patients with IS, specific social support measures may include structured psychoeducation interventions, family caregiver empowerment programs, peer support and social integration, and multidisciplinary team coordination with community resources (Winstein et al., 2016; Clark et al., 2020). Consequently, evaluating patients’ perceived social support and enhancing their actual social support networks should be regarded as a strategy for preventing and managing cognitive decline in elderly IS patients.

## 5 Conclusion

This research, utilizing latent variable growth models, demonstrates that the general cognitive function of elderly IS patients shows a linear decline pattern. Cognitive change trajectories were classified into three categories: High Cognitive Function-Slow Decline Group (C1), Low Cognitive Function-Intermediate Decline Group (C2), and Low Cognitive Function-Rapid Decline Group (C3). Subsequent research revealed that age, serum Hcy concentrations, temporal lobe infarction, NIHSS scores, and MSPSS scores significantly affected cognitive change trajectories in elderly IS patients. Healthcare professionals can develop personalized intervention plans based on the characteristics of different categories and their influencing factors to delay cognitive decline in patients and enhance their quality of life. Furthermore, universal interventions and targeted interventions for high-risk populations are not mutually exclusive but complementary strategies. In resource-constrained settings, our research provides objective evidence for identifying high-risk patients most in need of intensive interventions and implementing stratified management, aiming to optimize the cost-effectiveness of healthcare resources.

## 6 Limitations

Due to the lack of an age-matched control group of elderly individuals without IS, the overall cognitive decline trajectory we observed represents the combined effect of IS events and age-related changes occurring within 6 months. Therefore, this study cannot precisely quantify the independent net effect of IS itself relative to normal aging. However, the primary objective and core contribution of this research lies in revealing heterogeneous trajectories within the IS patient population and identifying their predictive factors. Our findings strongly suggest that even within this complex interaction, cognitive outcomes among elderly IS patients are far from uniform, exhibiting predictable and markedly divergent developmental patterns. Furthermore, while the 6-month follow-up period covers the critical diagnostic window for PSCI, extended follow-up would clarify the long-term trajectory of these patterns.

## Data Availability

The raw data supporting the conclusions of this article will be made available by the authors, without undue reservation. The data collection for this study was conducted as part of the Joint Research Project of the Health Commission of Shanghai Pudong New Area (PW2020D-6), a multicenter collaborative project involving multiple institutions, including Shuguang Hospital Affiliated to Shanghai University of Traditional Chinese Medicine, Shanghai Pudong New Area Gongli Hospital, and Shanghai Pudong New Area Traditional Chinese Medicine Hospital. The subsequent data analysis, interpretation, and manuscript drafting and revision were completed at Shanghai Pudong New Area Guangming Hospital of Traditional Chinese Medicine after the corresponding author and first author had joined this institution.
