# Correction: Analysis of cognitive function trajectories and influencing factors in elderly patients with ischemic stroke: a prospective longitudinal study

**DOI:** 10.3389/fnagi.2026.1959507

**Published:** 2026-08-18

**Authors:** Xinhao Chen, Chengxia Wei, Danli Zhao, Chenmei Zhou, Wenjing Wang, Gendi Lu

**Affiliations:** 1 Department of Neurology, Shanghai University of Traditional Chinese Medicine Affiliated Shanghai Pudong New Area Guangming Hospital of Traditional Chinese Medicine, Shanghai, China; 2 Department of Neurology, Shuguang Hospital Affiliated to Shanghai University of Traditional Chinese Medicine, Shanghai, China; 3 Department of Nursing, Shanghai University of Traditional Chinese Medicine Affiliated Shanghai Pudong New Area Hospital of Traditional Chinese Medicine, Shanghai, China

**Keywords:** cognitive function, influencing factors, ischemic stroke, latentvariables, trajectory

In the published article, due to an oversight during the submission process, the **Data availability statement** was incomplete. The **Data availability statement** was erroneously given as “The raw data supporting the conclusions of this article will be made available by the authors, without undue reservation.” The corrected **Data Availability statement** appears below.

“The raw data supporting the conclusions of this article will be made available by the authors, without undue reservation. The data collection for this study was conducted as part of the Joint Research Project of the Health Commission of Shanghai Pudong New Area (PW2020D-6), a multicenter collaborative project involving multiple institutions, including Shuguang Hospital Affiliated to Shanghai University of Traditional Chinese Medicine, Shanghai Pudong New Area Gongli Hospital, and Shanghai Pudong New Area Traditional Chinese Medicine Hospital. The subsequent data analysis, interpretation, and manuscript drafting and revision were completed at Shanghai Pudong New Area Guangming Hospital of Traditional Chinese Medicine after the corresponding author and first author had joined this institution.”

In the published article, due to an oversight during the submission process, two funding sources were unintentionally omitted from the **Funding** statement. The funders “Joint research project of Pudong New Area Health Commission, [PW2020D-6] to GL” and “Shuguang Hospital Nursing Research Fund Project, [2022SGHL05] to XC” were erroneously omitted. The corrected **Funding** statement appears below.

The author(s) declared that financial support was received for this work and/or its publication. This study was funded by the Clinical Research Project initiated by investigators from the Pudong New Area Health Commission of Shanghai Municipality (2026-PWDL-33) and the Nursing Science Xinglin Peak Research Program Cultivation Project of Shanghai University of Traditional Chinese Medicine (SHUTCMHLPF202411). The study was also supported by the Joint research project of Pudong New Area Health Commission, [PW2020D-6] awarded to GL, and the Shuguang Hospital Nursing Research Fund Project, [2022SGHL05], awarded to XC.

The original version of this article has been updated.

